# Assessing Mupirocin Resistance in MRSA Isolates in Hospitals in Cleveland, OH and Detroit, MI

**DOI:** 10.1017/ash.2024.272

**Published:** 2024-09-16

**Authors:** Taylor Yakubik, Collin Telchik, Andrea Grimbergen, Chetan Jinadatha, Munok Hwang, Hosoon Choi, Curtis Donskey, Jennifer Cadnum, Sorabh Dhar, Piyali Chatterjee, Keith Kaye

**Affiliations:** Baylor Scott and White; Department of Internal Medicine, Baylor Scott and White Medical Center, Temple, Texas; Baylor Scott & White; Central Texas Veterans Health Care System; Central Texas VA Research Foundation; Cleveland VA Medical Center; Harper Unive Hosp; Rutgers Robert Wood Johnson Medical School

## Abstract

**Background:** Methicillin-resistant Staphylococcus aureus (MRSA) is a common pathogen responsible for nosocomial and community-acquired infections with high morbidity and mortality1. MRSA nasal colonization is a major risk factor for developing infection in the hospital setting2,3. Decolonization of MRSA carriers is a strategy to decrease recurrence or to prevent new MRSA infections3,4. Decolonization with nasal mupirocin 2% and chlorhexidine baths has been shown to decrease the risk of MRSA infection after hospital discharge3. Mupirocin is an RNA synthetase inhibitor with activity against MRSA5. Resistance of MRSA isolates to mupirocin has been described previously6. As topical disinfectants play a crucial role in prevention of MRSA infection in a variety of settings, it is important to monitor the emergence of resistance. The goal of this study was to determine the prevalence of mupirocin resistance among MRSA samples isolated from two different regions in the United States (U.S). **Methods:** Our study had a total of 474 MRSA samples that were obtained from hospitals in Detroit, MI (287 samples) and Cleveland, OH (187 samples). After whole genome sequencing using NextSeq (Illumina Inc., CA) platform the data was analyzed using ResFinder 4.1, to identify antimicrobial resistance which can be either acquired or chromosomally mediated mutations. To visualize the presence of genes of interest the resistance genes were tallied on a spread sheet. **Results:** Mupirocin resistance gene was detected in five of 287 (1.74%) MRSA samples from the Detroit hospitals, all of which were associated with the mupA gene. Samples collected from the Cleveland area hospital demonstrated mupirocin resistance in seven samples of 187 (3.74%), all associated again with the mupA gene. One sample from the Detroit group showed resistance to both mupirocin and chlorhexidine. **Conclusions:** Prevalence of mupirocin resistance gene varied between the two hospital locations. Resistance to mupirocin has been documented in association with mutations in the mupA gene as well as chromosomal point mutations that can lead to either low or high-level resistance7,8. Although the mechanisms are not fully clear, mupA gene has been associated with high-level resistance9. Mupirocin resistance among MRSA isolates has increased over time9. MRSA infections remain an important etiology of nosocomial and community-acquired infections and common practice to combat this issue is universal decolonization with mupirocin10. It is critical to understand and monitor for development of mupirocin resistance as mupirocin remains one of the most effective tools to prevent invasive infection with MRSA in many patient populations.

